# Supplementary material for: Behaviour change interventions improve maternal and child nutrition in sub-Saharan Africa: A systematic review
Source: PLOS Glob Public Health. 2023 Mar 30;3(3):e0000401. doi: 10.1371/journal.pgph.0000401 (PMC10062616; doi:10.1371/journal.pgph.0000401)
Supplement: S1 Table — (DOCX) [file pgph.0000401.s001.docx]

# S1 Table: Behaviour change systematic review search strategy

**Search strategy**

**Databases**

Cochrane database, EMBASE, Medline, web of science, CINAHL, PSYCHinfo, scopus

**Inclusion criteria**

| Study Design | All study designs evaluating maternal and child nutrition interventions. |
| --- | --- |
| Population | At least one of the following populations living in sub-Saharan Africa:   - women of child bearing age (preconception) - pregnant women - fathers - infants, children and adolescents |
| Interventions | Nutrition related interventions |
| Comparator | Non behavioural change intervention |
| Outcomes | At least one of the following outcomes for:   - Health outcomes, including physical and psychological - Behavioural outcomes to nutrition - Both short and long term outcomes |

**Search terms**

| Population | (Sub Sahara* Africa OR subsahara* OR Africa South of the Sahara )  OR  (Angola OR Benin OR Botswana OR Burkina Faso OR Burundi OR Cameroon OR Cape Verde OR Central Africa OR Central African Republic OR Chad OR Comoros OR Congo OR Cote d*Ivoire OR Democratic Republic Congo OR Dijbouti OR Equatorial Guinea OR Eritrea OR Ethiopia OR Gabon OR Gambia OR Ghana OR Guinea OR Guinea Bissau OR Kenya OR Lesotho OR Liberia OR Madagascar OR Malawi OR Mali OR Mayotte OR Mozambique OR Namibia OR Niger OR Nigeria OR Rwanda OR Sahel OR Senegal OR Sierra Leone OR Somalia OR South Africa OR South Sudan OR Sudan OR Swaziland OR Tanzania OR Togo OR Uganda OR Zambia OR Zimbabwe )  AND  (**Matern*** OR Mother*OR **pregnan*** OR pre natal OR prenatal OR post natal OR **postnatal** OR **preconception** OR pre conception OR reproduc* OR paternal OR **parental** OR **family planning** OR 1000 days )  OR  (**adolescen*** OR youth OR young OR teen* OR student* OR girl* OR boy* OR pupil* OR **pubert* )**  OR  (offspring OR **infant*** OR **child** OR children OR **childhood** OR baby OR babies OR **newborn OR new born** OR paediatric* ) |
| --- | --- |
| Intervention | AND  ((**Nutrition*** OR Diet* OR food OR eat* OR nutrient OR feed*)  Adj3  (Attribution Theory OR Health locus of control OR Self determination theor* OR Risk perception OR self affirmation theory OR Self efficacy OR Stage of change model OR health action process approach OR Health belief model OR Theory of planned behav* OR social cognitive theor* OR Transtheoretical Model of Change OR Theory of Planned Behav* OR information Motivation Behav* skill* OR IBM OR Social Learning Theor* OR diffusion of innovation OR community mobilisation OR ecological perspective OR COMB OR COM B OR Capability, Opportunity, Motivation Behav* OR behav* change wheel))  OR  ((**Nutrition*** OR Diet* OR food OR eat* OR nutrient OR feed*)  Adj3  (social norm* OR attitude OR perception OR belief OR susceptibility OR autonomy OR health promotion OR public health campaign OR empower* OR resilien*OR goal setting OR implementation intention* OR co*production intervention))  OR  ((**Nutrition*** OR Diet* OR food OR eat* OR nutrient OR feed*)  Adj3  **(**behav* change technique* OR **behav* change** **theor*** OR behav* change OR behav* change intervention OR behav* modification OR community behav* change OR prevent behav* OR behav* change communication OR cultur* change* OR theoretical domain framework ))  OR  ((**Nutrition*** OR Diet* OR food OR eat* OR nutrient OR feed*)  Adj3  (**Health Psycholog*** theor* OR Health Psychology OR critical Psycholog* theor* OR clinical psycholog* theor* OR social Psycholog* theor* OR **cross cultural psycholog*** OR anthropology OR psychosocial OR biopsychosocial OR international psychology OR social science OR social medicine OR behav* medicine)) |
| Outcome | AND  ((**Nutrition*** OR Diet* OR food OR eat* OR nutrient OR feed*)  adj3  (**Health OR health*care OR wellbeing OR mental health OR psychological function* OR cognition** ))  OR  ((**Nutrition*** OR Diet* OR food OR eat* OR nutrient OR feed*)  adj3  (**human capital OR economic* OR social OR employment OR education** ))  OR  ((**Nutrition*** OR Diet* OR food OR eat* OR nutrient OR feed*)  adj3  (choice OR behav* OR habit* OR pattern OR prefer* OR selection OR portion OR size OR tast* OR binge OR comfort OR crav* OR perception OR perceive* OR belief OR attitude OR control OR norm*OR value* OR fruit* OR vegetable*)) |
