# Supplementary material for: Behaviour change interventions improve maternal and child nutrition in sub-Saharan Africa: A systematic review
Source: PLOS Glob Public Health. 2023 Mar 30;3(3):e0000401. doi: 10.1371/journal.pgph.0000401 (PMC10062616; doi:10.1371/journal.pgph.0000401)
Supplement: S2 Table — (DOCX) [file pgph.0000401.s002.docx]

# S2 Table: Behaviour Change systematic review screening document

| Study Characteristics | Inclusion criteria: | | Exclusion criteria: |
| --- | --- | --- | --- |
|  |  |  |  |
| Type of study | Intervention implementation (RCTs, cohort, longitudinal etc),  intervention evaluation  systematic reviews of interventions | | Protocols, commentary articles, conference papers, abstracts  Qualitative methodology to explore perceptions around nutrition  Surveys to investigate nutrition status |
| Participants | Populations living in sub-Saharan Africa:  women of child bearing age (preconception)  pregnant women  fathers  adolescents  new-borns  infants  children | | Populations living outside sub-Saharan Africa  Adults above child bearing age  Expats living in Africa |
| Types of intervention | Nutrition interventions with behavioural science component such as education, communication, social support etc  Nutrition interventions with psychological components such as personality, cognition, social psychology  Individual, family or community based | | Non-nutrition interventions  Interventions solely focused on HIV/AIDS  malaria or other infectious diseases  Breastfeeding  Interview/Focus group studies  Survey studies |
| Types of outcome measures | At least one of the following outcomes for infants, children and adolescents:  Nutrition outcomes  Behavioural outcomes  Short and long term health outcomes | |  |
| INCLUDE | | EXCLUDE | |
| Reason for exclusion |  | | |
| Notes: | | | |
