# Supplementary material for: Behaviour change interventions improve maternal and child nutrition in sub-Saharan Africa: A systematic review
Source: PLOS Glob Public Health. 2023 Mar 30;3(3):e0000401. doi: 10.1371/journal.pgph.0000401 (PMC10062616; doi:10.1371/journal.pgph.0000401)
Supplement: S3 Table — (DOCX) [file pgph.0000401.s003.docx]

# S3 Table: Behaviour Change Wheel (Michie et al, 2011)

| **Interventions** | **Definition** | **Examples** |
| --- | --- | --- |
| Education | Increasing knowledge or understanding | Providing information to promote healthy eating |
| Persuasion | Using communication to induce positive or negative feelings or stimulate action | Using imagery to motivate increases in physical activity |
| Incentivisation | Creating expectation of reward | Using prize draws to induce attempts to stop smoking |
| Coercion | Creating expectation of punishment or cost | Raising the financial cost to reduce excessive alcohol consumption |
| Training | Imparting skills | Advanced driver training to increase safe driving |
| Restriction | Using rules to reduce the opportunity to engage in the target behaviour (or to increase the target behaviour by reducing the opportunity to engage in competing behaviours) | Prohibiting sales of solvents to people under 18 to reduce use for intoxication |
| Environmental restructuring | Changing the physical or social context | Providing on-screen prompts for GPs to ask about smoking behaviour |
| Modelling | Providing an example for people to aspire to or imitate | Using TV drama scenes involving safe-sex practices to increase condom use |
| Enablement | Increasing means/reducing barriers to increase capability or opportunity | Behavioural support for smoking cessation in sub-Saharan Africa, medication for cognitive deficits, surgery to reduce obesity, prostheses to promote physical activity |
