# Supplementary material for: Behaviour change interventions improve maternal and child nutrition in sub-Saharan Africa: A systematic review
Source: PLOS Glob Public Health. 2023 Mar 30;3(3):e0000401. doi: 10.1371/journal.pgph.0000401 (PMC10062616; doi:10.1371/journal.pgph.0000401)
Supplement: S4 Table — (DOCX) [file pgph.0000401.s004.docx]

# S4 Table: Behaviour change systematic review quality assessment scoring rubric

|  | Item | Risk of bias | | |
| --- | --- | --- | --- | --- |
|  |  | Low (+1) | Medium (0) | High (1) |
| 1 | Study design | Randomised controlled trial, Quasi-experimental studies that include a control group | Evaluation of Randomised controlled trial such as process review | Experimental studies that do not use a control group. |
| 2 | Randomisation | Participants or communities are randomised in large clusters. |  | Not randomised, or inadequate randomisation methods such as birth date used. OR randomisation methods not described |
| 3 | Blinding | Participants and analysts were blinded to treatment condition. | Participants or communities were not sufficiently blinded, but analysis of results was blinded. | No blinding, or insufficient blinding methods. |
| 4 | Were groups similar at baseline? | Appropriate statistical tests (chi-square and/or t-tests) used to analyse differences between groups at baseline, and found that there were no significant differences. | Groups are similar, but there are some differences that are judged to be acceptable. | Analyst did not test for differences at baseline, or there were significant differences between groups. |
| 5 | Selection | Participants were randomly selected. | Community based interventions that require health professionals. | Volunteers are recruited. |
| 6 | Loss to follow-up | Loss to follow up of less than 10% AND similar loss in all groups. | More dropouts than expected (10%-30%). Similar loss between groups. | High dropout rate (>30%) and/or significant difference in follow up between groups. |
| 7 | Dietary assessment | Very reliable methodology that does not rely on self-report | Measures are reliable, but self-reported | Unreliable methods including questionnaires that have not been piloted or validated, and methods that increase the likelihood of socially acceptable responses |
| 8 | Behaviour change intervention component | Interventions clearly outlined a behaviour change or psychological theory within the intervention design | Components from the Behaviour Change Wheel can be associated to the intervention. | There was no or limited mention of behaviour change intervention functions |
| 9 | Performance bias | The intervention was delivered in a way that was not variable eg. Computer-generated. | The intervention was probably delivered consistently. Eg. The same person delivering a lesson to multiple groups. | The intervention was likely to have been delivered differently across the trial. Eg. Different health workers promoting nutrition education |
| 10 | Intention to treat | Intention to treat analysis was clearly used |  | Intention to treat was not used, or not stated |
| 11 | Analytic methods | Statistical tests used are rigorous and appropriate for the data set. (Regression modelling, ANOVA, etc) OR rigorous qualitative analysis (thematic analysis) | Statistical methods are effective, but not as rigorous as they could be. | Statistical methods are limited, and only report descriptive stats OR tests used are not appropriate for the dataset. |
| 12 | Did the analysis adjust for confounding?   - Educational attainment or SES - BMI/weight - Gender - Age - Baseline behaviour | The statistical model used adjusts for all relevant confounding factors. | The model adjusts for 3 or 4 confounders. | The analysis adjusts for fewer than 3 confounders |
