# Supplementary material for: Behaviour change interventions improve maternal and child nutrition in sub-Saharan Africa: A systematic review
Source: PLOS Glob Public Health. 2023 Mar 30;3(3):e0000401. doi: 10.1371/journal.pgph.0000401 (PMC10062616; doi:10.1371/journal.pgph.0000401)
Supplement: S5 Table — (DOCX) [file pgph.0000401.s005.docx]

# S5 Table: Behaviour change systematic review quality assessment form

|  | **Quality Assessment (Author, year, country, journal)** | | |
| --- | --- | --- | --- |
|  | Criteria | Description | Risk of bias score |
| 1 | Study design |  |  |
| 2 | Randomisation |  |  |
| 3 | Blinding |  |  |
| 4 | Were groups similar at baseline? |  |  |
| 5 | Selection (Were the selection criteria for the study specified?) |  |  |
| 6 | Loss to follow-up |  |  |
| 7 | Dietary assessment |  |  |
| 8 | Behaviour change intervention component |  |  |
| 9 | Performance bias -  Was the intervention executed in the same way across all participants/clusters? |  |  |
| 10 | Intention to treat |  |  |
| 11 | Analytical methods |  |  |
| 12 | Did the analysis adjust for confounding? |  |  |
|  | Reviewer’s comments |  |  |
|  |  |  | |
