# Supplementary material for: Behaviour change interventions improve maternal and child nutrition in sub-Saharan Africa: A systematic review
Source: PLOS Glob Public Health. 2023 Mar 30;3(3):e0000401. doi: 10.1371/journal.pgph.0000401 (PMC10062616; doi:10.1371/journal.pgph.0000401)
Supplement: Attachment — Submitted filename: BC review paper_PGPH_response to reviewers v1.1.docx [file pgph.0000401.s008.docx]

**Dr. Roopa Shivashankar**

PLOS Global Public Health

26^th^ July 2022

Dear Dr Shivashankar,

**Manuscript PLOS Global Public Health- PGPH-D-22-00527. ‘Behaviour change interventions improve maternal and child nutrition in sub-Saharan Africa: a systematic review’**

We thank the reviewers for their helpful responses to the manuscript and the editors for considering our manuscript. Below, we respond to the points raised by reviewers and have attached both a revised clean version of our manuscript and one with tracked changes as requested.

**Manuscript PLOS Global Public Health- PGPH-D-22-00527. ‘Behaviour change interventions improve maternal and child nutrition in sub-Saharan Africa: a systematic review’**

|  | **Comment** | **Author Response** | **Page No. of clean Manuscript** | **Text Excerpt** |
| --- | --- | --- | --- | --- |
| **Reviewer 1** | | | | |
| 1 | The manuscript discusses important issues in nutrition which are currently needed to address many nutrition problems in SSA and provide a mechanism for better approaches. The article registrar (PROSPERO) and use of others have been clearly mapped. Generally, paragraphing of the discussion section(from line 173) is poor, needs some review. Presently, paragraphs are too long and makes the reading difficult. | We thank the reviewer for their supportive review of this paper. We have reorganised the discussion section and made the paragraphs shorter. | P73-77 | **Discussion**  This review of 71 studies addressed the question of whether nutrition-specific and nutrition-sensitive interventions were more effective in improving maternal and child nutrition outcomes in sub-Saharan Africa if they included behaviour change functions. It was found that those that were based on behaviour change theory, counselling and communication, produced the most significant positive impacts on infant and children nutrition outcomes. This included reduced prevalence of wasting, underweight and stunting, and improved diet outcomes including dietary diversity and total food consumption. There is little evidence that these interventions improved maternal and infant nutrient intake. This could be because changes in nutrient intake were not often measured due to a lack of comprehensive data on the nutrient composition of foods. These interventions reviewed also identified improvements in maternal psychosocial outcomes in relation to nutrition, including knowledge, practice, attitude, intention, confidence, capability and wellbeing.  Interventions which included the Behaviour Change Wheel intervention functions, i.e. incentivisation, persuasion and environmental restructuring, tended to be those that improved maternal and child nutrition and psychosocial outcomes. The COM-B model suggests that not addressing participant’s motivation reduces the effectiveness of behaviour change interventions. This analysis in Table 5 demonstrates that nutrition-specific and nutrition-sensitive interventions cannot alone address motivation and require additional behaviour change functions in order to address behaviours that improve maternal and child nutrition and health outcomes.  **Strengths and limitations**  A strength of this systematic review is the inclusion of a wide variety of studies and using a range of intervention methods and outcomes. This provided a broad overview of the way in which behavioural functions may increase effectiveness of nutrition interventions to improve maternal and child nutrition outcomes in sub-Saharan Africa. One limitation was that breastfeeding interventions were excluded. This was due to previous systematic reviews finding limited evidence of behaviour change components in the interventions^(33, 34)^. One weakness of the review is that mainly papers written and published in English were reviewed. Another is that not all authors of these papers explicitly identified behaviour change functions in their descriptions of interventions, meaning that we may have missed other relevant papers. All titles, abstracts and full texts were double-screened against the inclusion and exclusion criteria, however, in order to minimise this possibility. Consistency in the types of data extracted and in judgements of the quality of the studies was ensured by having two independent reviewers review the included studies. **Implications**  Improving nutritional outcomes requires behaviour change. To change nutrition behaviour, the COM-B model suggests that capability, opportunity and motivation should all be addressed^(28)^. Our analysis demonstrates that most nutrition-specific and nutrition-sensitive interventions only address participants’ capability and opportunities. Nutrition interventions therefore require the addition of behaviour change functions such as behaviour change communication and counselling to address participants’ automatic and reflective motivation. Automatic motivation is defined as the unconscious process that “energizes and directs behaviour” ^(28)^(p4), including habits and emotional responses. Reflective motivation describes conscious processes such as analytical decision-making.  For example, one trial in Burkina Faso gave women land and seedlings (physical opportunity) and also had volunteers facilitate women’s groups using behaviour change communication that was empowering and motivating (automatic and reflective motivation^(28)^). Education functions of the communications in the trial in Burkina Faso may have changed the conscious decisions women were making about how to feed themselves and their children (reflective motivation) ^(54-56)^. The groups may have also created new social norms that encouraged women to change their dietary habits (automatic motivation)..  This review linked three Behaviour Change Wheel intervention functions to improved nutrition and psychosocial outcomes. These were incentivisation, persuasion and environmental restructuring. All are hypothesised to change motivations^(28)^. Providing incentives, which include cash transfers and food vouchers, has been found to improve health outcomes. This could be because it increases household disposable income allowing families to spend more money on food^(61, 107)^. This is particularly significant in improving outcomes in the poorest communities. Another incentive used was nutritional supplements, which increased intervention attendance and uptake^(63)^. In contrast, failing to incentivise community health workers was given as a reason for their lack of motivation for delivering interventions^(60)^. Overall, there is compelling evidence that incentives can be a powerful motivating force in behaviour change interventions^(113)^.  Persuasive communications were a common feature of many of the interventions in this review. . These tended to focus on improving diet quality and diversity and were delivered through singing groups, storytelling and women’s groups. The Behaviour Change Wheel indicates that persuasion, because it is often emotive and induced positive or negative feelings, engages both reflective and automatic motivations^(28)^. This distinguishes it from purely educational communications which work by informing conscious decision-making (reflective motivation).  In one intervention that included persuasive communication, grandmothers were encouraged through nutrition education to promote improved nutritional practices related to pregnancy and infant feeding within their families^(103)^. This was delivered through songs, storytelling and group discussions and made an emotional appeal to grandmothers’ intrinsic commitment to family well-being. These persuasive communications were therefore explicitly designed to induce an emotional response (automatic motivation) as well as educate and inform (reflective motivation).  Environmental restructuring is the process of changing the physical and social environment, which is hypothesised to indirectly change motivation by cuing behaviour in a way which is often unconscious (automatic motivation). In the context of this review, environmental restructuring referred to provision of land, seedlings, livestock and equipment with the intention of increasing the effectiveness of community food production for both consumption and sale^(54-56, 64, 106)^. It can be speculated that this provision addresses automatic motivations by increasing availability and diversity of food in these communities and supporting creation of new eating habits intended to improve diet quality.  Figure 4 illustrates the way in which all these intervention functions can be drawn together. Encouragingly, the addition of behaviour change functions to nutrition interventions may provide good value for money. An economic analysis within an intervention in Burundi calculated the relative cost of behaviour change functions accounted for approximately 13% of the budget whereas provision of food rations took up 30%^(52)^. Longitudinal studies are required to assess the impact of nutritional outcomes across children’s lifecourse and as communities undergo economic, nutritional, and societal transition^(114)^. |
| **Reviewer 2** | | | | |
| 3 | The article meets the requirements proposed by journal and knowledge to the scientific community. The authors fulfilled the necessary methodological requirements. Therefore, I recommend the article for publication. | We thank the reviewer for their supportive feedback on this paper. |  | N/A |
| **Reviewer 3** | | | | |
| 5 | Thanks for sharing your manuscript for review!  I was delighted to read it and can see it was a lot of work.  I have few comments:  In your background section, please introduce and briefly describe the health psychology perspective that you intend to use for your analysis. | Thank you for the supportive feedback on the paper. We have introduced the health psychology perspective in the background and methods sections. | P5  P6 | Behavioural scientists and health psychologists argue that those designing behaviour change interventions should engage with the target population, understand their motivation to change, and adapt interventions to the contexts that facilitate change including environment and social networks^(26)^. One model used by Health Psychologists is the Behaviour Change Wheel (Figure 1) which encapsulates features applicable to intervention design and implementation^(28)^. It outlines nine behavioural intervention functions, that aims to address deficits in one or more of the three underlying human factors – **C**apability (physical and psychological), **O**pportunity (physical and social), and **M**otivation (automatic and reflective) that influence **B**ehaviour (COM-B model) (Figure 2).  Full texts were screened by a Health Psychologist to select only interventions that had a behaviour change component and analyse the findings using the Behaviour Change Wheel ^(28)^ (Appendix C) |
| 6 | Line 111/2 + 140/141: “Further studies were sought by hand-searching bibliographies of included 112 studies, tracking citations on Google Scholar, and asking experts on the topic”. This is not reflected on the PRISMA chart. Also, chart is figure three in text and figure one in appendix. Please revise for consistency. | Thank you, we have updated the PRISMA chart to account for consulting experts and tracking citations and references of included papers. The chart is figure three on the document. | Figure 3 | Figure 3 |
| 7 | Line 132: Data analysis section need further details. Please provide information about how the analysis was done and data synthesised. At present, not clear if this was narrative synthesis. It reads more as qualitative description! | Thank you, we have added more information about the data analysis and synthesis. | P8 | **Data analysis**  Study designs were coded using an established method^(37)^ based on the functions from the Behaviour Change Wheel and coded to the COM-B model^(28)^. This is a well-evidenced behaviour change framework and this type of analysis has been carried out in other research studies^(28)^. Each intervention was reviewed independently by two trainee Health Psychologists who identified which Behaviour Change Wheel intervention functions were included in each intervention. The results section was structured around the main nutrition, psychosocial and behavioural outcomes, as advised by a nutritionist expert. Studies that included one of these outcomes were described under the appropriate heading. Findings are described using a narrative synthesis approach^(38)^. |
| 8 | Line 153/4: can you please provide further explanation as to how the finding could be informative? | We have updated the sentence to show how the findings can be informative even with a high risk of bias. | P9 | Studies were not removed if they were deemed low quality as findings might still have been informative providing novel evidence on using behaviour change and nutrition components together in an intervention, accepting that there was some bias in the intervention design. This was accounted for in the analysis and discussion of this paper. However, all findings are presented alongside their risk of bias to highlight which studies’ findings may be less credible. The studies deemed as low quality are not given as much weight in this review due to the possible bias and accuracy of results and interpretation. |
| 9 | Table 3: very informative table! Please proofread as still has typos e.g. row 1 “The intervention significant* increased consumption of ..” | Thank you. We have been through the table and corrected any typos. | Table 3 | Table 3 |
| 10 | Line 173 Anthropometric measurement, please consider summarising this section (and the subsequent results sections) around the findings most pertinent to your aim. At present, you are presenting too many results. | Thank you for this feedback. We have re-summarised the results section to focus it more aligned to the aims. | P66-70 | **Anthropometric markers**  A mother and child lipid supplementation RCT, graded as medium quality, combined with maternal nutrition counselling in Madagascar reported an increase in infants’ length-for-age z-scores (LAZ) of 0.210–0.216SD and stunting reduced by 8.2–9.0% compared to the control^(20, 75)^. Two similar medium-low-quality interventions in Malawi found mixed results, where one intervention had no effect on infant LAZ scores or stunting prevalence, but both interventions found significantly higher weight-for-length z-score (WLZ) and mid-upper arm circumference at 18 months to three years follow-up^(77, 78, 82, 85)^. These results have to be viewed with caution, however, since the trial was low quality being not randomised or blinded.  Two high-quality studies of interventions that provided food rations or vouchers and behaviour change communication to mothers in Burundi and Ethiopia, showed reduced prevalence of child stunting in the intervention compared to the control arm^(48-52, 70, 111)^. Behaviour change communication and unconditional cash transfers intervention which was deemed medium quality, to mothers in Togo reduced low birthweight (<2,500g) and protected infant height-for-age z-scores (HAZ) in the intervention arm compared to HAZ decline in the control arm, but had no impact on stunting^(61)^.  An agricultural production and behaviour change communication intervention for mothers in Burkina Faso, the study of which was rated high quality, significantly improved child wasting, diarrhoea, haemoglobin levels and rates of anaemia, especially among the youngest, as well as reducing underweight prevalence^(54, 56, 109)^. One chicken production intervention, rated high quality, improved infant HAZ and weight-for-age z-scores (WAZ) both with and without behaviour change communication in Ethiopia^(64)^. A RCT, graded high quality, that provided families with eggs and behaviour change communication significantly decreased the prevalence of infant wasting and underweight^(74)^. An agricultural (tools and agricultural extension workers), behaviour change communication and nutrition counselling intervention, graded high quality, in Ethiopia reduced prevalence of stunting (36.3% to 22.8%) in children aged 6–23 months^(67, 68, 76)^. One RCT, graded high-quality, that trained local Tanzanian women and farmers to deliver agricultural education to households as well as them receiving seedlings did not find significantly improved infant HAZs and WHZs scores^(72)^.  **Dietary outcomes**  Two behaviour change communication interventions, graded high quality, increased maternal dietary diversity, and the proportion of children consuming >4 food groups in Burundi^(48, 49, 51, 52, 111)^, and egg consumption in Ethiopia^(79-81)^. One behaviour change communication RCT, deemed medium-quality, found increased infant consumption of milk, but this was not significant^(69)^. One RCT of an intervention providing behaviour change communication and food rations, rated as high quality, increased energy consumption in pregnant women and mothers from Burundi^(48, 49, 51, 52, 111)^.  Two interventions, graded as medium-high quality and based on nutrition education, found no significant changes in the nutrient intake of children in Malawi and South Africa^(44, 88, 89)^. Yet one RCT rated as medium-quality found significant infant dietary diversity improvement with a combined nutrition education and counselling approach in Malawi^(71)^. Interventions graded medium quality that included nutrition education and cooking demonstrations increased diet diversity scores of women and children in Ethiopia^(42)^ and South Africa^(88, 89)^. One intervention underpinned by Social Cognitive Theory and the Theory of Planned Behaviour, graded high-quality, had school children in South Africa meeting 5-a-day fruit and vegetable guidelines^(45, 46)^.  Two low-medium rated quality psychosocial interventions increased pregnant women’s and mothers’ dietary diversity, and household food consumption in the Central African Republic^(107)^ and Ethiopia^(79-81)^. A intervention graded medium-quality that delivered radio dramas on maternal nutrition in Ghana found improved infant dietary diversity^(86)^. One RCT, deemed high-quality, that trained local Tanzanian women and farmers to deliver agricultural education to households as well as them receiving seedlings found significantly improved infant dietary diversity and food insecurity^(72)^.  **Psychosocial outcomes**  Interventions aimed to address psychosocial outcomes including nutrition knowledge (n=17)^(40-42, 44-46, 58, 61, 71, 73, 84, 90, 94, 96, 97, 103, 104)^, practices (e.g. food/recipes preparation)(n=5)^(42, 90, 94, 96, 98)^, attitudes (n=6)^(42, 45, 46, 62, 90, 94)^, intentions (n=4)^(45, 46, 62, 99)^ and confidence in eating healthy food (n=1)^(104)^. These were variously underpinned by behaviour change theory(n=10)^(42, 45, 46, 58, 62, 84, 90, 94, 96, 98)^, strategies to increase self-efficacy (n=5)^(40-42, 87, 99)^, behaviour change communication (n=2)^(44, 61)^ or behaviour change techniques (n=1)^(97)^.  In two interventions rated high quality and one rated low quality, women’s capability to feed themselves and their children more nutritious food increased, including interventions based on behaviour change communication in Burkina Faso^(54, 56, 109)^ and Zambia^(83, 110)^, and a psychosocial intervention in Ghana^(91)^. Five medium-high rated quality psychosocial interventions achieved positive psychosocial outcomes, which improved caregiver feeding behaviours. This included increasing women’s psychological well-being in the Central African Republic^(107)^ and Uganda^(53)^. Caregivers of children with cerebral palsy in Tanzania improved their feeding skills^(70)^. Caregiver relationships and communication improved leading to children’s prosocial behaviour in Mozambique^(65)^. Mother’s expression of love and responsiveness during feeding interactions increased in South Africa^(66)^. An intervention graded medium-quality which delivered radio dramas on maternal nutrition in Ghana found increased mother’s knowledge of feeding practices^(86)^. Qualitative evaluations identified barriers to the success of behavioural interventions including availability of, and access to, nutritious and affordable foods and less time for mothers to care for children^(106)^. |
| 11 | Line 274 issues with references | Amended. | P70 | Interventions were coded on the basis of involving one or more Behaviour Change Wheel intervention functions (Figure 1)(28). |
| 12 | Discussion: very good concepts introduced, I think it will benefit from reorganisation to communicate points clearer. | We thank the reviewer for their helpful comment and have reorganised the discussion section to make the points more clearly. | P73-77 | **Discussion**  This review of 71 studies addressed the question of whether nutrition-specific and nutrition-sensitive interventions were more effective in improving maternal and child nutrition outcomes in sub-Saharan Africa if they included behaviour change functions. It was found that those that were based on behaviour change theory, counselling and communication, produced the most significant positive impacts on infant and children nutrition outcomes. This included reduced prevalence of wasting, underweight and stunting, and improved diet outcomes including dietary diversity and total food consumption. There is little evidence that these interventions improved maternal and infant nutrient intake. This could be because changes in nutrient intake were not often measured due to a lack of comprehensive data on the nutrient composition of foods. These interventions reviewed also identified improvements in maternal psychosocial outcomes in relation to nutrition, including knowledge, practice, attitude, intention, confidence, capability and wellbeing.  Interventions which included the Behaviour Change Wheel intervention functions, i.e. incentivisation, persuasion and environmental restructuring, tended to be those that improved maternal and child nutrition and psychosocial outcomes. The COM-B model suggests that not addressing participant’s motivation reduces the effectiveness of behaviour change interventions. This analysis in Table 5 demonstrates that nutrition-specific and nutrition-sensitive interventions cannot alone address motivation and require additional behaviour change functions in order to address behaviours that improve maternal and child nutrition and health outcomes.  **Strengths and limitations**  A strength of this systematic review is the inclusion of a wide variety of studies and using a range of intervention methods and outcomes. This provided a broad overview of the way in which behavioural functions may increase effectiveness of nutrition interventions to improve maternal and child nutrition outcomes in sub-Saharan Africa. One limitation was that breastfeeding interventions were excluded. This was due to previous systematic reviews finding limited evidence of behaviour change components in the interventions^(33, 34)^. One weakness of the review is that mainly papers written and published in English were reviewed. Another is that not all authors of these papers explicitly identified behaviour change functions in their descriptions of interventions, meaning that we may have missed other relevant papers. All titles, abstracts and full texts were double-screened against the inclusion and exclusion criteria, however, in order to minimise this possibility. Consistency in the types of data extracted and in judgements of the quality of the studies was ensured by having two independent reviewers review the included studies. **Implications**  Improving nutritional outcomes requires behaviour change. To change nutrition behaviour, the COM-B model suggests that capability, opportunity and motivation should all be addressed^(28)^. Our analysis demonstrates that most nutrition-specific and nutrition-sensitive interventions only address participants’ capability and opportunities. Nutrition interventions therefore require the addition of behaviour change functions such as behaviour change communication and counselling to address participants’ automatic and reflective motivation. Automatic motivation is defined as the unconscious process that “energizes and directs behaviour” ^(28)^(p4), including habits and emotional responses. Reflective motivation describes conscious processes such as analytical decision-making.  For example, one trial in Burkina Faso gave women land and seedlings (physical opportunity) and also had volunteers facilitate women’s groups using behaviour change communication that was empowering and motivating (automatic and reflective motivation^(28)^). Education functions of the communications in the trial in Burkina Faso may have changed the conscious decisions women were making about how to feed themselves and their children (reflective motivation) ^(54-56)^. The groups may have also created new social norms that encouraged women to change their dietary habits (automatic motivation)..  This review linked three Behaviour Change Wheel intervention functions to improved nutrition and psychosocial outcomes. These were incentivisation, persuasion and environmental restructuring. All are hypothesised to change motivations^(28)^. Providing incentives, which include cash transfers and food vouchers, has been found to improve health outcomes. This could be because it increases household disposable income allowing families to spend more money on food^(61, 107)^. This is particularly significant in improving outcomes in the poorest communities. Another incentive used was nutritional supplements, which increased intervention attendance and uptake^(63)^. In contrast, failing to incentivise community health workers was given as a reason for their lack of motivation for delivering interventions^(60)^. Overall, there is compelling evidence that incentives can be a powerful motivating force in behaviour change interventions^(113)^.  Persuasive communications were a common feature of many of the interventions in this review. . These tended to focus on improving diet quality and diversity and were delivered through singing groups, storytelling and women’s groups. The Behaviour Change Wheel indicates that persuasion, because it is often emotive and induced positive or negative feelings, engages both reflective and automatic motivations^(28)^. This distinguishes it from purely educational communications which work by informing conscious decision-making (reflective motivation).  In one intervention that included persuasive communication, grandmothers were encouraged through nutrition education to promote improved nutritional practices related to pregnancy and infant feeding within their families^(103)^. This was delivered through songs, storytelling and group discussions and made an emotional appeal to grandmothers’ intrinsic commitment to family well-being. These persuasive communications were therefore explicitly designed to induce an emotional response (automatic motivation) as well as educate and inform (reflective motivation).  Environmental restructuring is the process of changing the physical and social environment, which is hypothesised to indirectly change motivation by cuing behaviour in a way which is often unconscious (automatic motivation). In the context of this review, environmental restructuring referred to provision of land, seedlings, livestock and equipment with the intention of increasing the effectiveness of community food production for both consumption and sale^(54-56, 64, 106)^. It can be speculated that this provision addresses automatic motivations by increasing availability and diversity of food in these communities and supporting creation of new eating habits intended to improve diet quality.  Figure 4 illustrates the way in which all these intervention functions can be drawn together. Encouragingly, the addition of behaviour change functions to nutrition interventions may provide good value for money. An economic analysis within an intervention in Burundi calculated the relative cost of behaviour change functions accounted for approximately 13% of the budget whereas provision of food rations took up 30%^(52)^. Longitudinal studies are required to assess the impact of nutritional outcomes across children’s lifecourse and as communities undergo economic, nutritional, and societal transition^(114)^. |
